# The Utility of Next-Generation Sequencing in the Treatment Decision-Making for Metastatic Non-Small-Cell Lung Cancer

**DOI:** 10.7759/cureus.16919

**Published:** 2021-08-05

**Authors:** Maria Cristina Orlov-Slavu, Ana Maria Popa, Adrian Tulin, Anca Pantea Stoian, Catalina Poiana, Cristian Paleru, Valentin Calu, Cornelia Nitipir

**Affiliations:** 1 Oncology Department, Carol Davila University of Medicine and Pharmacy, Bucharest, ROU; 2 General Surgery, Agrippa Ionescu Emergency Hospital, Bucharest, ROU; 3 Human Anatomy Department, Carol Davila University of Medicine and Pharmacy, Bucharest, ROU; 4 Diabetes, Nutrition and Metabolic Diseases Department, Carol Davila University of Medicine and Pharmacy, Bucharest, ROU; 5 Endocrinology, National Institute of Endocrinology C.I.Parhon, Bucharest, ROU; 6 Thoracic Surgery, Marius Nasta National Institute of Pneumology, Bucharest, ROU; 7 General Surgery, Elias Emergency Hospital, Bucharest, ROU

**Keywords:** cancer genomic profiling, targeted therapy, oncology, tumor agnostic treatment, next-generation sequencing

## Abstract

Next-generation sequencing (NGS) is a fast and relatively inexpensive method to sequence a large number of genes with crucial importance in cancer medicine. Nowadays, NGS is frequently used in diagnostic and therapeutic decisions in oncology; however, recently, it was demonstrated that only a few cancer sites actually benefit from this assessment. Moreover, the association of a mutant gene with a targeted drug is not always as predicted during in-vitro trials and is often not associated with tumor response. To predict the efficacy of such an association several classification systems have been developed. The present review aims to analyze the most important tumor agnostic treatment trials and assess how they shape selecting cancer patients for NGS. Moreover, it aims to determine how mutation-drug associations can be classified by their targetability and level of evidence of efficacy in non-small-cell lung cancer.

## Introduction and background

Next-generation sequencing (NGS) is the most accessible modality to describe genomic alterations in patients with cancer from both tumor tissue samples and circulating cell-free DNA. The characteristics of each tumor and possible drug targets can be determined using NGS. Tumor tissue samples can be inadequate for biomarker analysis because the DNA extracted is insufficient and/or degraded. To overcome these limitations, researchers have explored the possibility of using liquid biopsy as an alternative method for biomarker testing. The possibility to personalize oncological treatment is thought to lead to superior efficacy than classical drug targets. However, detecting targetable mutations is not always followed by therapeutic success, and the same drug may not have the same results in different cancer sites and histologies [1].

In order not to use these costly genetic assessments in vain, the classification of genetic alterations by their targetability has been proposed. Practicing oncologists report low self-confidence when choosing a type of drug-alteration association, as has been reported in the literature [2].

The present review aims to describe the most important trials centered on personalized oncology and tissue agnostic therapies, to describe how eligible patients for these tests can be selected, and to determine how one alteration and its correspondent drug can be classified according to the targetability and level of evidence of efficacy in non-small-cell lung cancer (NSCLC).

## Review

Insights gained from important precision medicine trials

We consider the National Cancer Institute-Molecular Analysis for Therapy Choice (NCI-MATCH) and SHIVA (molecularly targeted therapy based on tumor molecular profiling versus conventional therapy for advanced cancer) to be the landmark trials for histology agnostic cancer treatment. These trials have shaped how we view precision medicine today [3,4].

To better illustrate this some of the data presented in these two trials will be presented here. In the NCI-MATCH trial, 65 patients with 45 different histologies were selected to receive the phosphoinositide 3-kinase (PI3K) inhibitor taselisib. All of the selected patients had the *phosphatidylinositol-4,5-bisphosphate 3-kinase catalytic subunit alpha* (*PIK3CA*) activator mutation, but no tumor response was recorded [5]. In addition, patients with solid tumors or lymphomas harboring *BRAF* mutations other than the well-known *V600E* did not benefit from trametinib treatment in the same trial [6]. Moreover, when all *human epidermal growth factor receptor 2* amplified gene patients (except gastric and breast cancer patients) were treated with ado-trastuzumab emtansine, no benefit was reported [7].

Second, we consider the data from the SHIVA trial, the first prospective trial that evaluated the efficacy of NGS-selected oncology treatments in heavily treated patients with different cancer sites. The trial enrolled 195 patients with alterations in rapidly accelerated fibrosarcoma-mitogen-activated protein kinase, PI3K, and hormonal receptor signaling pathways. The patients were randomized according to physician choice of cytotoxic chemotherapy or targeted treatment. The progression-free survival (PFS) was not superior in the experimental arm (2.3 months versus 2 months; hazard ratio = 0.88, confidence interval = 0.65-1.19, p = 0.41) [4].

Finally, the insight from these data can be summarized as the following: there was no reliable differentiation between the supposed driver mutations and other possible actionable mutations and the design of these trials was unidirectional and took into account only one treatment-alteration association. It would be ideal to analyze the entire mutational landscape, discuss the significance of all alterations, and prioritize their targeting. Even if not all tested patients have targetable mutations, the global data from this analysis can be very helpful in cancer research. These trials not only teach how to design future tumor agnostic therapy studies but also shape the way we view mutation targetability in general [8].

Tools to aid decision-making in precision medicine

To avoid these problems, classifications were developed to aid physicians in selecting targeted therapies for the alterations. These classifications organize the current knowledge about the efficacy of drugs in different alterations and histologies. All of them describe the antitumor effect analyzing the magnitude of clinical benefit. Chronologically, the first classification was published in 2014 by Van Allen et al. and included five levels of evidence that reported the predictive, prognostic, and diagnostic value of the biomarkers used to select therapies [9]. In 2015, Dienstmann et al. proposed a different ranking method that considered the genetic abnormality, the variant, and the cancer site in which it can be found and reported the magnitude of clinical benefit considering each of these [10]. In 2016, Sukhai et al. focused on somatic variants and proposed classification included the ability to be a driver mutation, the site it can be found in, and the harboring histologies [11].

The first ranking that related possible drugs with their level of evidence and how they are perceived in international guidelines, especially the National Comprehensive Cancer Network was published in 2017 and was called OncoKB. This classification includes the alteration-drug associations categorized into four levels of evidence and ranks them according to their acknowledgment by decision agencies, such as the Food and Drug Administration. In addition, it also takes into consideration that targeting one association does not have the same impact in different histologies, that some genetic abnormalities are predictive of resistance, and that some oncogenic mutations cannot be targeted [12].

However, the most used ranking system is the ESMO Scale for Clinical Actionability of molecular Targets (ESCAT) which links the genetic alteration to its clinical relevance. When establishing in which tier to classify a drug, clinical trials are analyzed for statistical significance. One example for different histologies is presented in Table 1. Tier I includes associations that are supported by proof from clinical trials and can be considered as the standard of care. Tiers IA, IB, and IC can be defined as the following: IA, randomized clinical trials that proved a benefit in survival; IB: randomized clinical trials that reported clinically meaningful benefit; and IC, efficacy proven in basket trials.

**Table 1:** ESCAT ranking in other histologies than NSCLC. AKT1: AKT serine/threonine kinase 1; ERBB3: Erb-b2 receptor tyrosine kinase 3; ESCAT: ESMO Scale for Clinical Actionability of molecular Targets; FGFR: fibroblast growth factor receptor; NSCLC: non-small-cell lung cancer; NTRK: neurotrophic tyrosine kinase; PIK3CA: phosphatidylinositol-4,5-bisphosphate 3-kinase catalytic subunit alpha;PTEN: phosphatase and tensin homolog

Tier level	Alteration	Tumor site	Drug used
IA	BRCA 1/2 germline mutation	Breast cancer	Talazoparib [13]
IB	FGFRfusion	Cholangiocarcinoma	Pemigatinib [14]
IC	NTRK fusion	Breast cancer, colorectal cancer, gastric cancer	Entrectinib [15,16]
IIA	PTEN deletion or mutation	Prostate adenocarcinoma	Ipatasertib(+abiraterone) [17,18]
IIB	AKT1 mutation	Breast cancer	AZD5363 [19]
IIIA	PIK3CA hotspot mutation	Hepatocellular carcinoma	Alpelisib [20]
IIIB	ERBB3 mutation	Breast cancer, gastric cancer	Neratinib [21]

Tier II describes associations that were reported as significant but are not sustained by prospective studies. When deciding to treat with such an association, the drug should be given in an access program or in an ongoing prospective trial. Tier IIA includes associations that were proven to be beneficial in retrospective studies, and IIB includes associations that cannot be subjected to a prospective analysis. Tier III includes alterations whose importance has been proven for other histologies than the one in question. In IIIA, the benefit has already been proven in other tumor types, whereas IIIB describes an association with an extremely probable clinical benefit with no proof of efficacy yet (for no histology). Tier IV describes genetic abnormalities that have been proven to be targetable in preclinical trials. Tier IVA includes proof from in-vitro studies or in-vitro cancer models, and IVB includes predictions of efficacy derived from informatic systems. Tier X includes all abnormalities that are known to be untargetable or have never been proven beneficial when associated with drugs. However, such abnormalities should not be permanently considered part of this subgroup (they could prove useful when associated with a different alteration or when tested with different technology) [22].

Next-generation sequencing for non-small-cell lung cancer: when and where?

**Figure 1:**
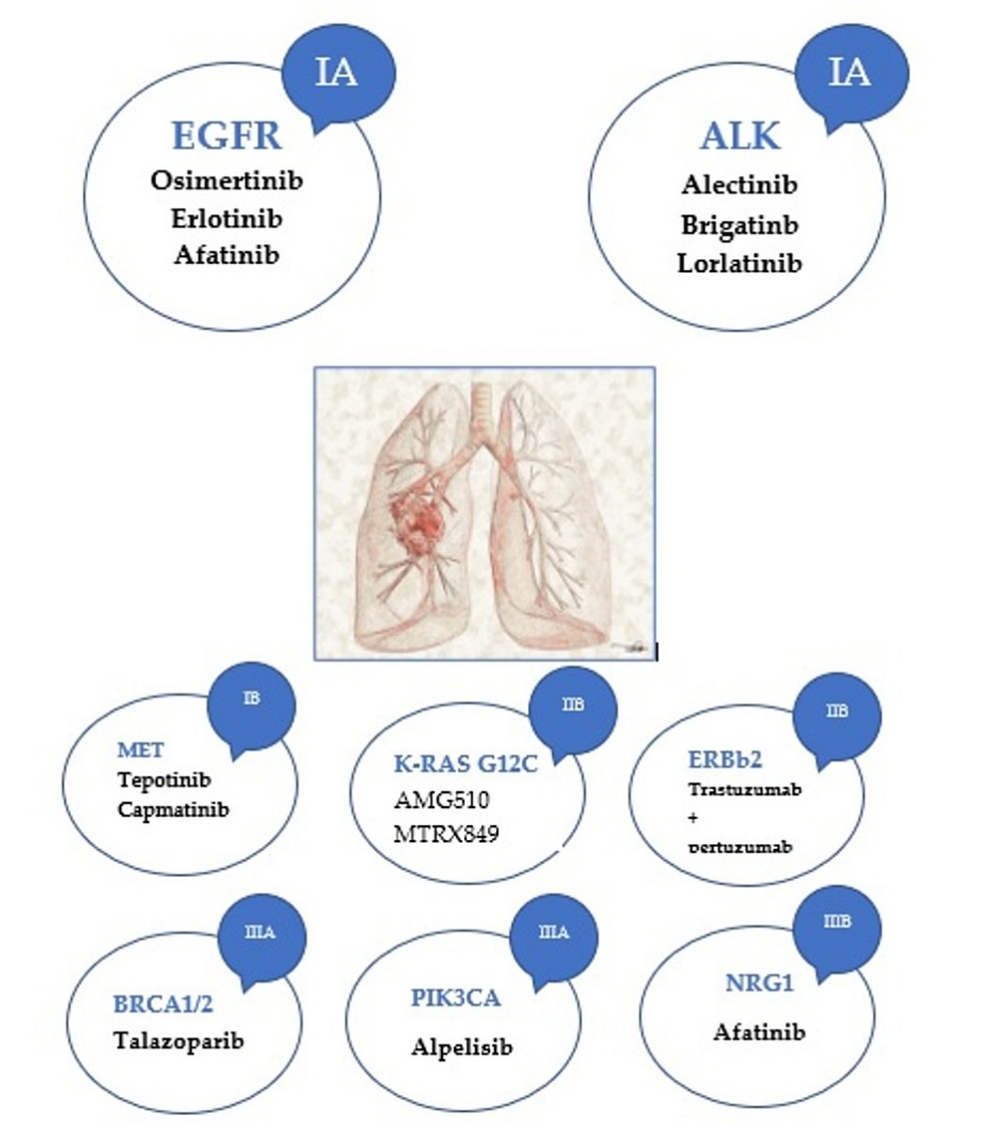
Genetic alterations in NSCLC and their level of evidence for targetability according to ESCAT ALK: anaplastic lymphoma kinase; EGFR: epidermal growth factor receptor; ERBb2: erythroblastic oncogene B; ESCAT: ESMO Scale for Clinical Actionability of molecular Targets; K-RAS: Kirsten rat sarcoma oncogene; NRG1: neuregulin 1 gene; NSCLC: non-small-cell lung cancer; PIK3CA: phosphatidylinositol-4,5-bisphosphate 3-kinase catalytic subunit alpha

The latest recommendations for NGS testing made by the European Society of Medical Oncology) list NSCLC as the only cancer in which it makes sense to use multigenic panels that include all alterations with ESCAT I level of targetability. Given that gene fusions are very important in this cancer site, the NGS panels used are of the DNA or the RNA type. The most important mutations and fusions are illustrated in Figure 1.

For global benefit, the tests for alterations with evidence lower than ESCAT 1 have debatable significance. They can be considered if the patient is negative for frequent alterations, the healthcare system in the country has a reimbursement plan for off-label drugs, or if there is an early access program in which patients can be enrolled to receive them [23].

The most common alterations in NSCLC are the well-known *epidermal growth factor receptor* (*EGFR*) mutation and *anaplastic lymphoma kinase* (*ALK*) fusion. Within the different subtypes of *EGFR* mutations, the levels of evidence for targetability vary. The most common driver mutations, namely, exon 19 deletion and exon 21 (L858R) substitution (15% of the general population and 50-60% of the Asian population), are associated with the most data. To these, the mutation *T790M* in exon 20 is added, which is usually acquired and marks resistance to treatment with older generation tyrosine kinase inhibitors such as erlotinib [24,25].

All these alterations are classified as ESCAT I, and the preferred treatments in these circumstances include osimertinib, erlotinib, afatinib, and gefitinib. Most countries reimburse at least one of these treatments as the first line in these patients [26-29]. However, there are *EGFR* mutations with a lower level of targetability such as *G719X* mutation in exon 18, *S768I* in exon 20, and *L861Q* in exon 21. The prevalence of these mutations in NSCLC is 10% and can be classified as ESCAT IIB. Prospective, nonrandomized studies have shown benefit in at least PFS when patients with rare *EGFR* mutations were treated with osimertinib and afatinib [26,30-32].

Insertions in exon 20, with a prevalence of approximately 2% in NSCLC, are considered predictive of the lack of response to classical tyrosine kinase inhibitors used in patients with *EGFR* mutations. However, there is currently a therapeutic option in this situation: poziotinib. An ongoing phase 2 trial has already reported a response rate of approximately 64% with this treatment in patients with exon 20 *EGFR* insertions. ESCAT ranking for this alteration remains open until further data are obtained [33].

The next gene alteration in prevalence and importance in NSCLC is *ALK* fusion (5%), classified as ESCAT IA. The preferred treatment in this situation includes alectinib, brigatinib, or lorlatinib [34-36].

Among the alterations with a lower level of evidence for targetability, we mention *MET*, *BRAF*, *neurotrophic tyrosine kinase *(*NTRK*), *rearranged during transfection* (*RET*), *Kirsten rat sarcoma oncogene* (*K-RAS*), *ROS*, *erythroblastic oncogene B* (*ERBB2*), *BReast CAncer gene* (*BRCA1/2*), *PIK3CA*, and *neuregulin 1 gene* (*NRG1*). Phase I and II clinical trials have confirmed clinical benefit in targeting and improving overall response rate [23].

The next alteration worth reviewing is *MET* exon 14 skipping mutation considered targetable with crizotinib, capmatinib, and tepotinib, with the latter two being considered by current guidelines as preferred treatments. It has a level of evidence Ib and a frequency of 3% in NSCLC [23,37-39].

*K-RAS* can also be classified among the important mutations in NSCLC, being a relatively frequent mutation (12%). It has a level IIB targetability [23]. The most druggable subtype of this mutation is *K-RAS G12C* (substitution of glycine 12 to cysteine). Trials are underway with several drugs such as AMG510 (Amgen) and MTRX849 (Mirati Therapeutics) in both monotherapy or combined with immunotherapy [40,41]. In addition, some phase I trials with mature data included older drugs for *K-RAS* mutation. Two worth mentioning are sorafenib, a multikinase inhibitor that targets upstream of the substrate of this mutation combined with the mTOR inhibitor (mammalian target of rapamycin) and everolimus. Unfortunately, this combination was not enough to induce a lasting tumor response [42,43].

Among the alterations with lower levels of targetability in NSCLC, but worthy of special attention, are ESCAT IIB level: *ERBB2* (amplifications or hotspot mutations); ESCAT IIIA: *BRCA1/2* and *PIK3CA* (low frequency, approximately 1.2%); and *NRG 1*-ESCAT fusion IIIB [23]. The most important alterations in NSCLC and the proposed targeting drugs are illustrated in Figure 1.

The non-small-cell lung cancer issue: cost-effectiveness

Broad NGS testing is the most cost-effective in NSCLC of all histologies [44,45]. To analyze the financial aspects of NGS in NSCLC, two trials were selected with similar endpoints but different populations. The first one, by Mateo et al., included 174 patients with metastatic NSCLC and tested them for 29 DNA alterations using an NGS panel (the panel included* EGFR*, *BRAF*, *ERBB2*, *TP53*) and three ARN fusions, namely, *ALK*, *ROS*, *RET*. It aimed to determine the cost-effectiveness by analyzing the proportion of patients who would not have had detected the gene alteration had it not been for this test by reporting the actual financial burden and the time needed until the results were available [46]. However, the data can be considered biased as patients were only of Singaporean nationality, for which the percentage of *EGFR*-positive patients is higher than that for other populations. The trial concluded that NGS is a cost-efficient method to decide treatment in metastatic NSCLC and supported the implementation of NGS as a standard in the Asian population [46].

However, when NGS was compared with the classic sequencing test (the one including only *EGFR* mutations, *ALK* translocation, and programmed death-ligand 1), only 1% of patients benefited from the additional detection of alterations [47,48].

Steuten et al. compared the cost-effectiveness and clinical efficacy of single genetic marker tests with the determination of classical alterations in metastatic NSCLC by NGS. Cost-effectiveness was classified as moderate (after analyzing the cost of genetic tests, the frequency of targetable alterations, and the availability of correspondent therapies). Of the 5,688 patients studied, only 875 were tested by NGS. Overall, 21% received targeted treatment compared to 19% of the patients who underwent classical determination [49].

Overall, both trials reported moderate cost-effectiveness in the use of NGS in advanced NSCLC. To this must be added the default longer time to results. In addition, and most importantly, the use of NGS may lead to a situation where the patient has, according to the literature and guidelines, the indication of a targeted treatment, but it is not reimbursed in the country. These situations must be foreseen by public health forums of each country, eventually proposing a customized solution.

## Conclusions

To conclude, NGS testing in NSCLC for ESCAT IA genetic alterations is of paramount importance and should be considered standard. However, alterations with lower evidence targetability should always be considered, with careful consideration given to the financial and medical aspects involved.
